# Diagnostic Approach to Pneumonia in Immunocompromised Hosts

**DOI:** 10.3390/jcm14020389

**Published:** 2025-01-09

**Authors:** Nadir Ullah, Ludovica Fusco, Luigi Ametrano, Claudia Bartalucci, Daniele Roberto Giacobbe, Antonio Vena, Malgorzata Mikulska, Matteo Bassetti

**Affiliations:** 1 Department of Health Sciences (DISSAL), University of Genoa, 16126 Genoa, Italy; nadir.ullah@uvas.edu.pk (N.U.); bartalucciclaudia@gmail.com (C.B.); anton.vena@gmail.com (A.V.); m.mikulska@unige.it (M.M.); matteo.bassetti@hsanmartino.it (M.B.); 2 UO Clinica Malattie Infettive, IRCCS Ospedale Policlinico San Martino, 16126 Genoa, Italy; ludo940f@gmail.com (L.F.); luigi.ametrano@outlook.com (L.A.); 3 Department of Clinical Medicine and Surgery, University of Naples Federico II, 80138 Naples, Italy

**Keywords:** immunocompromised patients, pneumonia, microbiological diagnosis, culture, antigenic tests, polymerase chain reaction

## Abstract

In immunocompromised patients, pneumonia presents a diagnostic challenge due to diverse etiologies, nonspecific symptoms, overlapping radiological presentation, frequent co-infections, and the potential for rapid progression to severe disease. Thus, timely and accurate diagnosis of all pathogens is crucial. This narrative review explores the latest advancements in microbiological diagnostic techniques for pneumonia in immunocompromised patients. It covers major available microbiological tools for diagnosing both community-acquired and hospital-acquired pneumonia, encompassing a wide spectrum of pathogens including bacterial, viral, fungal, and parasitic. While traditional culture methods remain pivotal in identifying many pneumonia-causing etiologies, their limitations in sensitivity and time to results have led to the rise of non-invasive antigen tests and molecular diagnostics. These are increasingly employed alongside cultures and microscopy for more efficient diagnosis, mainly in viral and fungal infections. Lastly, we report the future of pneumonia diagnostics, exploring the potential of metagenomics and CRISPR/Cas13a for more precise and rapid pathogen detection in immunocompromised populations.

## 1. Introduction

Pneumonia is an acute infection of the lung parenchyma caused by different pathogens. [1]. The clinical presentation of pneumonia could be severe leading to intensive care unit (ICU) admission, especially in immunocompromised patients who are at high risk for hypoxemic acute respiratory failure and sepsis [2].

The American Thoracic Society (ATS) provides a clear definition and diagnostic criteria for immunocompromised host pneumonia (ICHP) [3]. ICHP is defined as infectious pneumonia occurring in individuals with a quantitative or functional impairment of host immune defenses. The diagnostic criteria for ICHP include clinical suspicion of a lung infection, with or without compatible clinical signs and symptoms, accompanied by radiographic evidence of a new or worsening pulmonary infiltrate [3].

While immunocompromised states can be innate, acquired immunodeficiency is currently becoming more prevalent due to recent advances in cancer chemotherapy, solid organ transplants (SOTs), hematopoietic cell transplantation (HCT), the use of immunomodulatory drugs, and the acquired immune deficiency syndrome (AIDS) [4]. Patients with immunosuppressive conditions are at higher risk of pneumonia, which may account for 75% of all pulmonary complications [4]. Various pathogens, including bacteria, viruses, fungi, and parasites, could be responsible for pneumonia in immunocompromised patients, although bacteria represent the leading cause followed by viruses [5,6].

Patient’s risk for infection with specific pathogens is influenced by the nature of their underlying immune defect (e.g., the increased susceptibility to encapsulated bacterial infections caused by *Streptococcus pneumoniae* or *Haemophilus influenzae* in patients with humoral immunity defects), but mixed immune defects (e.g., occurring in HCT recipients) should always be considered. Moreover, specific risk factors associated with the underling disease or its treatment can lead to an increased incidence in fungal, mycobacterial, and viral infections [5].

International guidelines for managing lung disease in critically ill immunocompromised patients emphasize the importance of obtaining valid diagnostic samples with invasive and non-invasive investigations [7]. Various microbiological methods can be used to diagnose bacterial pneumonia. Culture methods of samples such as blood, sputum, and bronchoalveolar lavage fluid (BALF) should be employed, although diagnostic performance of these tests could be affected by the quality of the sampling, the specific pathogen involved (e.g., atypical respiratory pathogens), and prior antibiotic use [8,9]. Therefore, important diagnostic methods also include antigen and molecular testing, especially for viral infections in which polymerase chain reaction (PCR) is actually the reference standard diagnostic method [10]. Indeed, multiple respiratory viral and bacterial infections can be diagnosed simultaneously within 2–3 h using PCR-based diagnostic panels [11]. Regarding fungal pneumonia, cultures have low sensitivity, and the fungal growth can be slow. For these reasons, antigen-based diagnostics (in blood, BAL, and serum) is the cornerstone of diagnosis in immunocompromised patients, in addition to specific radiological findings, with molecular methods slowly gaining importance [12].

The purpose of this narrative review is to discuss microbiological diagnostic methods used in pneumonia in immunocompromised patients. This review article is organized as follows: (i) diagnostics methods for community-acquired bacterial pneumoniae (CABP) and hospital-acquired bacterial pneumonia (HABP), including ventilator-associated bacterial pneumonia (VABP); (ii) diagnostic methods for viral pneumonia; (iii) diagnostic methods for fungal pneumonia; (iv) potential future approaches to the diagnosis of pneumonia; (v) conclusions.

## 2. Diagnostic Methods for Bacterial Pneumonia

### 2.1. Community-Acquired Bacterial Pneumoniae

Among the most frequent bacterial pathogens responsible for community-acquired bacterial pneumonia (CABP) in immunocompromised patients are *Streptococcus pneumoniae*, *Staphylococcus aureus*, *Hemophilus influenzae*, *Klebsiella pneumoniae*, *Mycoplasma pneumoniae*, *Chlamydia pneumoniae*, and *Legionella pneumophila* [13].

Patients with severe-CABP (defined according to ERS/ESICM/ESCMID/ALAT guidelines as a CAP that require intensive care unit admission and might require organ support, encompassing almost 5% of patient with CAP) [14], require an extensive diagnostic approach, and different microbiological tests are available including traditional culture-based methods, microscopy, antigenic testing (e.g., on urine for *Streptococcus pneumoniae* and *Legionella pneumophila*), and molecular methods [15,16]. Blood cultures are recommended in the diagnostic work-up for severe-CABP and can be useful for achieving etiological diagnosis (except for atypical respiratory pathogens), although their sensitivity remains suboptimal. Thus, the diagnosis of pneumonia should not rely on blood cultures only, and their use should be carefully examined, especially with rising healthcare costs [17,18].

The diagnostic performance of various tests for CABP has been investigated across several studies. A Gram strain performed on respiratory specimens (mainly sputum) could be a rapid and useful tool to evaluate a bacterial cause of pneumonia. Its sensitivity could vary depending on the pathogen involved, reaching 62.5% for *Streptococcus pneumoniae*, 60.9% for *Haemophilus influenzae*, and just 9.1% for staphylococcal pneumonia, while reported specificity was 100% for staphylococcal pneumonia, 95.1% for *Haemophilus influenzae*, and 91.5% for *Streptococcus pneumoniae* in a prospective study conducted by Fukuyama and colleagues [19]. For instance, in a recent study, different traditional culturing methods (sputum Gram staining, BALF Gram staining, and sputum culture) were evaluated for diagnosis of a single bacterial pathogen among 287 children with CAP. Using a BALF culture as reference, the sensitivity and specificity for these methods were as follows: sputum Gram staining: sensitivity 70% and specificity 23%, sputum culture: sensitivity 64% and specificity 70%, and BALF Gram staining: sensitivity 60% and specificity 71% [20].

Respiratory specimen cultures remain crucial for performing phenotypical susceptibility testing and for the identification of etiological agents not detected by molecular methods, such as rapid direct respiratory panels. However, some important limitations should be acknowledged, such as low sensitivity and long turnaround time, while reduced sensitivity in the case of prior antibiotics can also affect the diagnostic accuracy of this method [8]. Specifically, for pneumococcal pneumonia, the sensitivity of sputum cultures has been reported to vary widely, from 29% to 94% [15].

Regarding antigenic testing, Sordé and colleagues evaluated the urinary pneumococcal antigen test in hospitalized patients with CABP, 20.3% of whom were immunocompromised, reporting a sensitivity of 70.5% and a specificity of 96% [21]. A meta-analysis of UAT performance in streptococcal pneumonia yielded similar results, with pooled sensitivities of 66% [95% confidence interval [CI], 62–71] for inpatients and 67% [95% CI 56–76] for mixed populations and specificities of 89% [95% CI 82–93] and 90% [95% CI 79–96], respectively. However, specificity was generally lower in studies involving immunocompromised patients [22]. On the other hand, a possible advantage of this test is the minimal impact of prior antibiotic use on diagnostic performance compared to respiratory cultures, as showed by Said and colleagues in a metanalysis [23].

Immunocompromised patients are particularly vulnerable to *Legionella pneumophila*, especially when cell-mediated immunity is compromised [24]. Certain clinical signs, such as diarrhea, elevated transaminases, hyponatremia, and increased LDH levels, along with environmental exposure, should raise suspicion for *Legionella* pneumonia, though symptoms are often nonspecific but rapidly progressive [25,26]. The diagnostic approach should include a urinary antigen test (that showed a pooled sensitivity and specificity of 79% [95% CI 71–85] and 100% [95% CI 99–100], respectively, in a meta-analysis), with additional tests such as respiratory PCR, when possible, since the urinary antigen test detects only *Legionella pneumophila* serotype 1 [27,28].

Regarding serological tests, they have been classically used for the etiological diagnosis of *Mycoplasma pneumoniae* CABP, although lower sensitivity than more recent molecular methods has been suggested [29]. Overall, molecular approaches can increase pathogen detection rates, as showed by an observational study including 323 patients with CABP in which the achieved pathogen detection rate was 87% with molecular methods compared to 39% [127/323 patients] with culture-based methods, helping in the antibiotic de-escalation approach after the results [30]. However, it is important to note that molecular testing includes a limited number of targets; thus, clinicians should always consider that some organisms (depending on the employed panel) cannot be detected, or the detected microorganism can also represent a respiratory tract colonization [30].

Diagnosis of opportunistic pathogens such as *Nocardia* spp. requires high suspicion and high pre-test probability, because the culture should be performed on dedicated media and have a longer turnaround time [31]. In this setting, molecular methods such as *Nocardia* spp.-specific PCR could be useful in establishing the diagnosis. A multicenter study evaluated a PCR-based assay for nocardiosis, reporting a sensitivity of 88% [7/8 patients] and a specificity of 74% [35/47 patients] in respiratory samples [32].

Finally, the risk of active tuberculosis is increased in patients with immune impairments including HIV, diabetes, cancer, hematological malignancies (HMs) or SOT, and those receiving systemic steroids or TNFα inhibitors, and a specific diagnostic work-up should be considered in a high-risk patient with a compatible clinical pattern [33]. The microbiological diagnosis of pulmonary tuberculosis is based on detecting mycobacteria through acid-fast staining, cultures, and molecular assays from induced sputum samples (three samples for both smears and cultures should be collected) or from a single lower-respiratory tract specimen in the presence of lung lesions. Indeed, the performance of these tests depends also on the form of pulmonary tuberculosis, with miliary TB having no connection with the bronchial system and thus exhibiting very low sensitivity in sputum or even BALF samples, as opposed to the cavitary form, with possible high load of mycobacteria. A meta-analysis of 27 studies reported the performance of PCR in sputum samples (culture as a reference test), with an overall sensitivity of 89% (95% CI, 85–92) and specificity of 99% (95% CI, 98–92), with higher sensitivity in the case of smear-positive vs. smear-negative samples: 98% (95% CI, 97–99) vs. 67% (95% CI, 60–74) of samples [34]. A higher sensitivity of 80% (95% CI 52–96) was also reported [35]. Nontuberculous mycobacteria (NTM) are species of mycobacteria other than *Mycobacterium tuberculosis* and *Mycobacterium leprae* who share similar risk factors for disseminated disease as those for tuberculosis, including HIV infection, steroid use, TNF-α inhibitors, diabetes, cancer, and SOT. Since NTM are environmental organisms, their presence in nonsterile respiratory specimens does not necessarily indicate a pathogenic role in lung disease, unlike in the case of tuberculosis [36]. According to the American Thoracic Society (ATS) and the Infectious Disease Society of America (IDSA), the diagnostic criteria for NTM lung disease include pulmonary symptoms, compatible radiographic findings, and either two positive sputum cultures, one positive BALF sample, or a positive lung biopsy culture with suggestive histological features [36]. In cases of disseminated disease, blood cultures on special media should be performed and maintained in incubation for at least 6 weeks. Bone marrow, fluid, or tissue samples from suspected sites should be sent for culture and histological examination with specific stains [36].

### 2.2. Hospital-Acquired Bacterial Pneumonia and Ventilator-Associated Bacterial Pneumonia

Conventional tests for diagnosing HABP and VABP include a Gram stain, blood culture, and culture of respiratory specimens [37]. Blood cultures are strongly recommended in all HABP and VABP episodes, although only a few cases are bacteremic, with reported low sensitivity (5–15%) across several studies [38,39,40], thus showing a suboptimal performance as a standalone diagnostic test [41].

Regarding cultures of respiratory specimens, a sputum culture should be performed in HABP if an adequate sample, characterized by a Gram stain showing few or no squamous epithelial cells, can be obtained [41]. In immunocompromised patients, however, lower respiratory tract specimens (e.g., collection of BALF fluid) are generally recommended for diagnosing HABP and VABP. A prospective study assessing the diagnostic performance of BALF cultures in HABP patients found that BALF cultures successfully identified 18 of 23 cases (78%), with infections defined by colony counts of at least 10^4 CFU/mL [42].

Molecular methods, particularly multiplex polymerase chain reaction (mPCR) syndromic panels, are increasingly utilized for the etiological diagnosis of HABP and VABP. A prospective study by Peiffer-Smadja and colleagues evaluated the diagnostic performance of mPCR on BAL and plugged telescoping catheter (PLC) samples from ICU patients with HABP/VABP. The mPCR panel used (Unyvero panel) demonstrated a specificity of 99% [95% CI 99–100] and sensitivity of 80% [95% CI 73–88], with higher sensitivity for Gram-negative bacteria (90% compared to 62% for Gram-positive cocci) [43]. Similar studies using the same panel reported sensitivities ranging from 73% [95% CI 59–84] to 88.8% and specificities between 94.9% and 97.9% [95% CI 95–99], using conventional culture methods as the reference standard [44,45]. A diagnostic meta-analysis found that, for the etiological diagnosis of methicillin-resistant *Staphylococcus aureus* (MRSA) in patients with HABP and CABP, the pooled sensitivity and specificity of PCR were 85% [95% CI 60–96] and 92.1% [95% CI 82–97], respectively. For MRSA VABP, the specificity was 93.7% [95% CI 77–98], but sensitivity was significantly lower at 40.3% [95% CI 17–68] [46]. Moreover, a PCR/electrospray ionization-mass spectrometry (PCR/ESI-MS) assay applied on BALF samples can provide quantitative data that may aid in differentiating between infection and colonization; although, this distinction should be based on the overall clinical picture, not just PCR results [47].

## 3. Diagnostic Methods for Viral Pneumonia

While an upper respiratory tract viral infection typically causes a self-limiting illness in an immunocompetent individual, it can lead to significant morbidity and mortality in an immunocompromised host. The severity of illness in this population is often attributed to the frequent occurrence of secondary infections with bacteria, fungi, or other viruses, as well as the potential spread of the virus to the lower respiratory tract [47].

While in the past, diagnostic methods for respiratory viruses include direct fluorescent antibody (DFA) assays, enzyme immunoassays, and viral culture, PCR is currently the most frequently used and most useful method, due to excellent sensitivity and specificity. Rapid Antigen Detection Tests (RADTs) have the advantage of lower cost, and the possibility of point-of-care use with immediate results. While their sensitivity for SARs-CoV-2 was acceptable, it is usually low for other viruses such as influenza or RSV [48,49].

A large study involving 6090 participants reported the performance of DFA and Rapid Antigen Detection Tests (RADTs) for the diagnosis of novel H1N1 influenza A virus along with other influenza subtypes. A total of 518/3789 (13.7%) positive results for all influenza A strains (seasonal H1N1, H3N2, and novel H1N1) were obtained with the RADT; 397/3271 (12.1%) with DFA tests. The sensitivity of the RADT and DFA assay for novel H1N1 were 21.2% and 47.2%, while the specificities were 99.5% for the RADT and 99.6% for the DFA assay [48]. A systematic review and meta-analysis reported the performance of the RADT for RSV virus infection, including 71 articles. The study reported a pooled sensitivity and specificity of 80% (95% CI 76–83) and 97% (95% CI 96–98), respectively; however, the sensitivity was 81% (95% CI 78–84) in children and only 29% (95 CI 11–48) in the adult population [49].

Viral cultures, although more sensitive, require several days to yield results and dedicated facilities, making them impractical for clinical use [50].

Real-time PCR (RT-PCR) can be used to detect various viruses from samples such as nasopharyngeal swabs, sputum, endotracheal aspirates, or BALF. Multiplex assays, which usually include influenza virus types A and B, respiratory syncytial virus (RSV), parainfluenza viruses (PIV), human rhinoviruses (HRV), human metapneumovirus (HMPV), and adenovirus (AdV), are highly sensitive and provide rapid diagnosis [51]. A review summarizing the diagnostic accuracies of RT-PCR for detecting influenza A and B, RSV, HMPV, and AdV reported that among the 5510 patient samples analyzed, the area under the receiver operating characteristic curve (AUROC) was 0.98 or higher for all the viruses mentioned, except for AdV, which had an AUROC of 0.89 [11]. Also, an automated nested multiplex PCR system, such as the FilmArray system, was found to detect up to 95% of viral pathogens with an average turnaround time of just 75 min [52,53]. Molecular methods are also recommended for the diagnosis of SARS-CoV-2 infection, since rapid antigen tests have lower sensitivity, especially in asymptomatic individuals [54].

Of note, if cytomegalovirus (CMV) is suspected, dedicated PCR should be performed, and in such cases, quantitative results are usually provided. Given the high sensitivity and negative predictive value, a negative CMV-DNA result in BALF can plausibly exclude CMV pneumonia, but there is no established cut-off for CMV-DNA in BALF that reliably differentiates between CMV pneumonia and pulmonary virus shedding without organ disease [55,56]. In a small prospective study involving 45 immunocompromised patients with lower respiratory tract infections, the authors assessed the incidence of CMV pneumonia and quantified plasma CMV DNA loads using a highly sensitive CMV nucleic acid amplification test [57]. A plasma CMV DNA load threshold of 2.91 log_10_ IU/mL was proposed as a potential screening tool to complement conventional methods for diagnosing CMV pneumonia in immunocompromised patients. They found a significant correlation between high plasma CMV DNA loads and elevated CMV DNA loads in BALF [57]. Indeed, blood testing for CMV and data on patient’s history and immune status might provide additional help in deciding if CMV pneumonia is likely.

In addition to CMV, also in case of AdV, testing of blood samples to detect viremia is also useful. In the case of AdV, viremia may occur and lead to dysfunction in a single organ system, such as interstitial pneumonia, or to disseminated infection affecting two or more organ systems, which significantly increases the mortality rate to between 8% and 26% [58]. Detection of AdV in the blood may precede symptomatic disease by 2 to 3 weeks, providing an opportunity for pre-emptive intervention in recipients of allogeneic HCT. PCR testing of blood specimens is recommended if AdV disease is suspected, as the presence of AdV DNA in the blood typically indicates disseminated infection [59]. Isolated respiratory infection can also occur, even in immunocompetent patients, and ADV pneumonia has been reported in healthy young adults. Identifying patients who could benefit from antiviral treatment remains a challenge in this setting, since the currently available anti-AdV antiviral (cidofovir) is associated with significant renal toxicity.

Finally, two aspects are important in case of viral pneumonia in immunocompromised patients. First, upper respiratory tract PCR results might already be negative in the case of viral infection that progressed to the lower respiratory tract. Therefore, in the case of clinical suspicion, PCR should be performed in BALF. Second, co-infections with different pathogens are very frequent in immunocompromised patients with pneumonia, and radiological patterns might be overlapping (e.g., tree-in-bud lesions). Therefore, comprehensive testing for the most frequent viral, bacterial, and fungal pathogens should be performed in BALF, and interpretation of the clinical impact of single pathogens might be challenging.

Last but not least, severe viral pneumonia, mainly due to influenza and SARS-CoV-2, was recognized as a significant risk factor for fungal pulmonary infection, mainly invasive aspergillosis.

## 4. Diagnostic Methods for Fungal Pneumonia

Among the fungi responsible for pneumonia in immunocompromised patients are molds (e.g., *Aspergillus* spp. and Mucorales), *Pneumocystis jirovecii*, and *Cryptococcus* spp. [31,60]. They are all airborne pathogens, and their spores can be inhaled from the air [31,61]. An invasive fungal disease (IFD) in immunocompromised patients can be classified as proven, probable, or possible according to the EORTC/MSGERC criteria [62]. Various microbiological diagnostic methods are available to help achieve a probable (and sometimes proven) diagnosis of fungal pneumonia in immunocompromised patients, including non-culture- and culture-based methods.

### 4.1. Microscopy, Culture, and Histology

Culture and microscopy are employed within the diagnostic algorithms for invasive pulmonary aspergillosis (IPA) and cryptococcal pneumonia, while *Pneumocystis jirovecii* cannot be cultured [63,64,65]. The reference standard for the proven diagnosis of *Pneumocystis jirovecii* pneumonia (PJP) involves microscopic examination of respiratory samples using dedicated staining methods [66,67].

For IPA, the identifications of *Aspergillus* spp. in a BALF culture defines a mycological criterion for the diagnosis of probable invasive aspergillosis, while a proven diagnosis can only be achieved through histology or positive culture from a sterile site, e.g., through lung biopsy [62,68]. Regarding proven diagnosis, due to bleeding risk or respiratory insufficiency, a lung biopsy is rarely performed in severely immunocompromised patients, such as those with hematological malignancies or HCT recipients. Therefore, diagnosis of IPA is more frequently documented as probable based on a culture of BALF or antigen tests, rather than proven. Regarding cultures, although their sensitivity is only around 50% for IPA, the isolation of *Aspergillus* spp., or any other filamentous fungus, from BALF culture provides important information on species identification and antifungal susceptibility [69,70,71]. For the diagnosis of PJP, *Pneumocystis jirovecii* cannot be cultured as reported above, so the proven diagnosis is defined through microscopy with specific staining methods, e.g., Grocott–Gomori methenamine silver stain (highest sensitivity reported), modified Papanicolaou, Wright–Giemsa, or Gram–Weigert stains [66]. It should also be considered that the fungal load may be different in different types of populations. For example, in the case of HIV-positive patients, the fungus load is usually high; whereas in HIV-negative patients, the fungus load is usually lower; thus, diagnostic accuracy could also be affected by these differences [72]. The induced sputum test with hypertonic saline is less invasive than BAL and has been successfully used in high-fungal-load infections but does not have a high sensitivity (reported between 55% and 90%) [73,74]. In fact, it has been reported that induced sputum samples had higher sensitivity in HIV-positive patients compared to HIV-negative patients [75,76,77]. A recent prospective study reported [9/18 patients] 50% sensitivity for microscopy by Grocott–Gomori methenamine silver staining for diagnosing PJP pneumonia in immunocompromised patients [78]. Overall, the major drawbacks of microscopic methods for diagnosing PJP are low sensitivity, dependence on the expertise of the mycologist, and the fact that they may be time-consuming for the laboratory.

Cryptococcal pneumonia is diagnosed through microscopy or cultures of blood and sputum or BALF, and *Cryptococcus* spp. typically grows within 2–3 days after specimen collection [31,79,80]. While susceptibility testing can be performed for *Cryptococcus* spp., its routine use is not recommended due to the limited evidence linking minimum inhibitory concentrations (MICs) with clinical outcomes [81].

Microscopy (both direct and as histopathology) and cultures of BALF or pulmonary biopsy samples are the cornerstones for the diagnosis of pulmonary mucormycosis [31,82,83]. Regarding culture methods, *Mucorales* spp. can grow within 1 to 7 days on fungal culture media, though cultures may only be positive in 50% of cases [83].

### 4.2. Antigen Testing

Antigen testing, such as serum and BALF galactomannan (GM) tests, is a cornerstone of the diagnosis of probable IPA and has been included for decades as a mycological criterion in the dedicated guidelines [84]. A single-center retrospective study reported 78.9% [95% CI 58–71] sensitivity of serum GM and 78.6% [95% CI 55–100] sensitivity of BALF GM for probable/proven IPA [85]; another retrospective study reported 93.2% [95% CI 86–97] sensitivity and 86.8% [95% CI 83–87] specificity for the BALF GM test for diagnosing proven/probable IPA [86]. The meta-analyses from Pfeiffer and colleagues and from Leeflang and colleagues reported 70–78% pooled sensitivity of serum GM for the diagnosis of invasive aspergillosis in hematological malignancy patients [87,88]. Another meta-analyses reported 89% [95% CI 83–93] sensitivity of BALF GM for IPA diagnosis in immunocompromised patients [89].

The sensitivity of GM is affected by the type of the tested population, with significantly higher sensitivity of serum GM in neutropenic patients. On the contrary, in SOT recipients the sensitivity of serum GM was reported to be as low as 22% [95% CI 3–60] [87], and limited data are available on the diagnostic performance of BALF in SOT. Another study reported a 100% sensitivity for BALF GM in diagnosing IPA in SOT recipients, whereas serum GM had a sensitivity of 25% [90]. However, it is important to note that the number of IPA patients in the study was very low (n = 5) [90].

Several causes of false positive GM results have been reported, with treatment with piperacillin/tazobactam being one of the most important [91]. Indeed, in a retrospective study that examined the relationship between three β-lactam antibiotics and the occurrence of false-positive GM test results in patients with HM, positive serum GM occurred in the case of treatment with 27 out of 39 batches of β-lactam antibiotics, and GM test results remained positive for up to 5 days after the termination of antibiotic treatment [92]. Nonetheless, although some residual GM might still be present in some piperacillin/tazobactam, the currently available formulations of piperacillin/tazobactam no longer appear to be responsible for false-positive GM results [93]. Interestingly, infections due to other fungi, such as *Fusarium* spp., *Penicillium* spp., or even *Histoplasma* spp., can also result in positive GM testing.

Another fungal biomarker, 1, 3 beta-d-glucan (BDG), is present in the cell wall of many pathogenic fungi (except for *Mucorales* spp. and cryptococci), and for this reason is not specific for invasive aspergillosis [31,94]. A systematic review on the comparison of PCR with serum BDG and GM from 2015, reported 76.9% [95% CI 66.7–84.8] and 89.4% [95% CI 87–91] sensitivity and specificity for serum BDG for IA diagnosis [95], with poor sensitivity of BDG in SOT recipients [96]. However, serum BDG was shown to be much more useful as a supporting diagnostic tool for PJP. A study reported that serum BDG in AIDS/HIV patients with pneumonia showed a sensitivity of 92.8% [95% CI 87–97] for PJP [97]. A meta-analysis conducted by Karageorgopoulos and colleagues reported 94.8% [95% CI 91–97%] sensitivity for the diagnosis of PJP in immunocompromised patients [98]. Another meta-analysis reported no differences in the specificity of the test while sensitivity was higher for HIV-positive patients and lower for HIV-negative patients [99].

The antigen method is a rapid method for the diagnosis of cryptococcal pneumonia. Serum, sputum, and BALF may be tested for the cryptococcal antigen. Oshima and colleagues assessed the diagnostic performance of cryptococcal glucuronoxylomannan antigen tests in BALF and serum in HIV-negative patients. They reported a BALF sensitivity of 82.6%, while the serum showed 73.9% sensitivity for diagnosing cryptococcal pneumonia in 23 confirmed cases [100]. Similarly to microscopy, it was reported that the serum cryptococcal antigen assay was less sensitive in HIV-negative immunocompromised patients with cryptococcal pneumonia than in HIV-positive patients with cryptococcal pneumonia, possibly due to lower fungal burden. More in detail, a sensitivity of 56–83% was reported in HIV-negative immunocompromised patients with cryptococcal pneumonia [79,101]. Indeed, while several studies reported very good diagnostic performance of the cryptococcal antigen test in HIV-positive patients with cryptococcal meningitis, the diagnostic performance of the cryptococcal antigen test in serum and BALF of patients with pulmonary cryptococcosis remains less clear [102].

Neither BDG nor GM are present in *Mucorales*; thus, negative results from these tests, in combination with lung computed tomography (CT) findings consistent with invasive fungal infection, may suggest mucormycosis and the need for dedicated diagnostic procedures such as a culture of BALF or lung samples [103]. Currently, there is no specific antigen test available for the diagnosis of *Mucorales* pneumonia, although some are under investigation, and molecular methods have been introduced [104,105].

### 4.3. Molecular Methods

PCR is a molecular non-culture test increasingly available for the diagnosis of various types of pulmonary fungal infections in immunocompromised patients [64]. With the development of new commercial assays and advancements in PCR standardization, the last EORTC/MSGERC definitions recognized PCR as a mycological criterion for probable IPA [64]. A meta-analysis by Han and colleagues analyzed the diagnostic performance of BALF PCR in immunocompromised high-risk patients, reporting an overall pooled specificity and sensitivity of 94% [95% CI 90–96] and 75% [95% CI 67–81], respectively, while for proven IPA, the specificity and sensitivity were 80% [95% CI 68–98] and 91% [95% CI 74–85] across 14 studies involving 2061 patients [106]. Another meta-analysis evaluated the diagnostic performance of PCR from whole blood or serum. When a single positive serum PCR result was required to diagnose IPA, the sensitivity and specificity were 80.5% [95% CI 73–86] and 78.5% [95% CI 68–86], respectively. However, when two consecutive positive PCR results were required, the specificity increased to 96.2% [95% CI, 90–99], while the sensitivity decreased to 58% [95% CI, 37–77] [107]. BALF PCR had high sensitivity but a possible lack of ability to differentiate airway colonization from infection, which is one of the limitations of PCR [64].

In addition to qualitative conventional PCR, nested PCR and quantitative real-time PCR (qPCR) are also available for diagnosing PJP. The performance of respiratory PCR assays for diagnosing PJP in high-risk patients, including HIV-positive and HIV-negative patients, was reported in a bivariate meta-analysis, with a pooled specificity and sensitivity for the overall population of 90% [95% CI 87–93] and 99% [95% CI 96–100], respectively [108]. Another meta-analysis reported the diagnostic performance of BALF PCR for the diagnosis of PJP in both HIV-positive and HIV-negative patients, with a pooled specificity and sensitivity of 91% [95% CI 83–96] and 98.3% [95% CI 91–99.7], respectively [109]. Since conventional PCR cannot differentiate airway colonization from true fungal infection [110], real-time PCR is more often recommended since its quantitative nature may help distinguish between airway colonization and true fungal infection; although, a clear cut-off still needs to be defined and standardized and may vary in different populations of immunocompromised patients [111,112]. Interestingly, in a prospective study of 86 HIV-positive patients with subacute respiratory symptoms who were suspected of having PJP, a qPCR based on the mtSSU gene from induced sputum samples was apparently able to potentially differentiate three groups (one group with PJP, one without PJP, and the third with colonized patients) based on quantification cycle (Cq) values; although, further external validation and standardization are warranted [113].

Molecular diagnostic tools have also been investigated for the diagnosis of cryptococcal pneumonia. A study utilized multiplex real-time PCR to detect *Cryptococcus* DNA in samples from AIDS patients with opportunistic pneumonia, including BALF, blood, biopsy, and other samples [114]. Multiplex Real-Time PCR was tested in 24 clinical strains and 43 clinical samples from 40 patients infected with HIV/AIDS having proven fungal infections. The in vitro sensitivity was 100% for clinical strains and 90.5% in clinical samples with positive results for 92.5% of patients, yielding positive results in four out of five cryptococcosis samples [114]. Additionally, PCR can distinguish *Cryptococcus* species by targeting the STR1F and STR1R genes [115].

For mucormycosis, molecular methods are particularly interesting since there are no antigenic methods available, and some studies have analyzed the identification of mucormycosis through PCR in serum and blood; although, this topic deserves further investigation [116]. In a small study, four cases of suspected pulmonary mucormycosis in patients with HM were successfully diagnosed using PCR on peripheral blood samples [117]. Another study employed qPCR for the early detection of mucormycosis in immunocompromised patients, detecting free DNA in the serum of 9 out of 10 patients earlier than the confirmed diagnosis through histopathological examination and positive culture, with the detection occurring between 3 and 68 days earlier [118].

### 4.4. Point-of-Care-Tests [POCTs] for Invasive Aspergillosis (IA)

Two main POCTs are now available for the diagnosis of IA, such as the *Aspergillus* Galactomannan lateral flow assay (GM-LFA) and *Aspergillus*-specific lateral flow device (AspLFD), while others are in development, and a body of evidence is growing rapidly [119]. Seven studies were published between 2020 and 2024: five [120,121,122,123,124] assessed the diagnostic performance of LFA, and two studies [125,126] covered AspLFD for IA diagnosis. The five studies on the LFA test reported 384 proven/probable/possible or chronic/probable IFD cases. Four of them used *Aspergillus* Galactomannan LFA (IMMY Diagnostics, Oklahoma, USA), and one study used QuicGM^TM^ *Aspergillus* Galactomannan LFA (Dynamiker Biotechnology [Tianjin] Co., Ltd., Tianjin, China). There were 68 proven/probable IA cases reported in two studies using the CE-marked AspLFD test.

#### 4.4.1. Aspergillus Galactomannan Later Flow Assay (LFA)

LFAs detect GM antigen in immunochromatographic tests and can provide rapid qualitative results in both serum and BALF samples. For both reported assays, quantitative results can be obtained through an automatic optical reader for the IMMY *Aspergillus* GM LFA and a fluorescence immunoassay analyzer for the Dynamiker LFA [119]. The details of these test have been recently reviewed [127].

A large multicenter study from China reported on 310 clinically suspected IA patients with various underlying conditions and evaluated the LFA assay as a screening tool in serum and BALF samples, with results interpreted through an immunoanalyzer and compared with the ELISA GM test. The sensitivity of the LFA was higher in BALF than serum samples (89% [95% CI 78–96] vs. 83% [95% CI 74–89]). Both the LFA and ELISA had similar specificity in serum and BALF samples, while the sensitivity of ELISA was higher in BALF than the LFA (93% [95% CI 82–98] vs. 89% [95% CI 78–96]). The total percent agreement between the LFA and ELISA in serum and BAL samples was 92% and 94%, while positive and negative percent agreement (PPA and NPA) for serum samples was 95% and 89%, with the discordance arising mainly due to the samples that were positive by ELISA and negative by the LFA [120].

Another prospective multicenter study from Turkey compared the performance of quantitative GM-LFA (using a cube reader for exact quantitate results) with GM-EIA in HM patients with suspected IA. Proven IA was diagnosed through sinonasal tissue culture or lung tissue biopsy. The sensitivity of the LFA test in serum samples was slightly higher in differentiating proven IA versus no IA than proven/probable IA versus no IA (83% vs. 75%), while specificity was 100%. Both GM-LFA and GM-EIA had 91% positive results in (10/11) proven/probable cases at a cut-off of 0.5, while for BALF an overall qualitative agreement of 82% between the two tests was observed [121].

Serin and colleagues compared serum GM LFA quantitative results to GM ELISA. They included 87 patients with IA: proven n = 11/87, probable n = 54/87, and possible n = 22/87. The test performance was reported for two groups (no IA = 76, and IA = 11). The LFA test demonstrated an excellent sensitivity and specificity of 91% for diagnosing proven IA, while GM ELISA had 0% sensitivity and 92% specificity, respectively [123].

Another study evaluated the performance of the GM-LFA in a cohort of mainly (82%) patients that had HM. Serum GM-LFA had a sensitivity and specificity of 97% [95% CI 94–99.5) 31/32] and 98% [95% CI 93–99.5] (98/100), respectively, in 32 patients with proven/probable or chronic IFD. When considering all samples, the sensitivity and specificity was 91%, and an overall qualitative agreement of 89% was observed between the two tests [122].

In a single-center retrospective study from the USA, 31 samples from 28 cancer patients with suspected IA were tested. The sensitivity and specificity of serum LFA-GM was 100% [95% CI 51–100] and 96% [95% CI 92–97] for proven IA, while EIA-GM had a lower sensitivity of 25% [95% CI 1.3–70] and specificity of 98% [95% CI 96–99] [124].

Mercier and colleagues reported in 2020 that empirical antifungal therapy significantly reduced the sensitivity of the LFA assay in BALF samples in patients with HM [128], while a recent multicenter study published in 2023 reported no significant difference in patients receiving antifungal prophylaxis or treatment compared to patients without treatment or prophylaxis. The sensitivity was 75% for all 83 patients with proven/probable IA, and after excluding 42 patients receiving antifungals (15 treatment and 27 prophylaxis), the sensitivity for proven/probable IA was 76% [121]. Limited data are available on the diagnostic performance of the LFA test in SOT recipients as compared to those with HM [129].

A multicenter study evaluated the performance of a CE-marked LFA test in patients with various underlying conditions. The sensitivity of the LFA test for diagnosing IA in BALF samples was excellent at 100% (10/10), while the specificity was very low, at only 17% (4/24) at a cut-off value of 0.5 for the optical density index (ODI). After increasing the cut-off value to 1.5 ODI, the specificity increased to 68%, while the sensitivity decreased to 68%, respectively [130].

The performance of the test differed by patients’ population and sample types (serum and BALF). A recent review published in 2024 [127] reported that sensitivity and specificity of LFA test in ICU patients having viral-associated pulmonary aspergillosis was 76% and 80% in BALF samples, while in the same population the sensitivity of serum samples dropped to 55%, and similar results were found in other studies in these setting, and diagnostic performance of the serum LFA-GM test was higher in neutropenic patients [127]. Finally, the meta-analysis from Zhang and colleagues also reported lower pooled sensitivity for the LFA test in serum samples as compared to BALF samples (71% [95% CI 56–87] vs. 83% [95% CI 72–94]) [131].

#### 4.4.2. Aspergillus-Specific Lateral Flow Device (AspLFD)

AspLFD is a qualitative immunochromatography test for the detection of the *Aspergillus* antigen in BALF and serum. The test uses a monoclonal antibody called JF5 to identify a glycoprotein (mannoproteins) that is generated by the *Aspergillus* species during active growth [127]. There are two recent studies on the diagnostic efficacy of CE-marked AspLFD for IA diagnosis, which included 279 patients with a wide variety of underlying conditions, including HM and other immunocompromised conditions.

One study compared the performance of the LFD to GM ELISA in 218 cancer patients (184 serum samples and 58 BALF). The overall performance of the test was excellent for diagnosing proven/probable IA vs. no IA (sensitivity 92% [95% CI 62–99.8] and specificity 95% [95% CI 91–98]). The sensitivity of the test was lower in serum samples as compared to BALF samples ([67% (95% CI 9–99] vs. 100% [95% CI 66–100]), while the specificity in serum samples was excellent at 99%. Both the LFD and GM had similar diagnostic performance in serum samples, while the LFD sensitivity was higher in BALF. Additionally, the study found that the sensitivity was higher in solid tumor patients as compared to patients with HM (100% [95% CI 69–100] vs. 50% [95% CI 1.3–99]) [125].

Another study from Hsiao and colleagues compared the performance of the LFD test with EIA GM in 91 immunocompromised patients, including 56 with HM and 35 on corticosteroids. The GM test had higher sensitivity than the LFD (90% (26/29) vs. 69% (20/29)), and a specificity of 99% [74/75] vs. 79% [59/75]; although, this finding was likely increased due to the use of the GM test for case classification. In addition, the study also found a significant difference in the discordance between LFD and GM in those who had been previously treated with AF (33%), as compared to those without such exposure (12%), using EORTC/MSGERC as the gold standard [126].

Finally, the diagnostic performance of the test can also vary based on population type and sample types. A review article from Heldt and Hoenigl evaluated the test performance in various settings, including SOT recipients, ICU patients, and other respiratory diseases patients. The review findings suggest that sensitivity is lowest in HM patients compared to the overall population (67% [36/54] vs. 73% [83/113]) [132].

The meta-analysis from Pan and colleagues from 2015 on LFD development studies reported lower pooled sensitivity for the LFD in serum samples as compared to BALF samples for diagnosing proven/probable IA in immunocompromised patients (68% [95% CI, 52–81] vs. 86% [95% CI 76–93]) [133].

A review article on POCTs reported on the diagnostic performance in BALF and serum samples for the AspLFD prototype and the CE-marked version. The sensitivity of the LFD prototype in BALF samples was high as compared to serum samples (71% [86/121] vs. 68% [30/44]), while the sensitivity of the test was lowest for patients with HM and highest for SOT recipients (69% [22/32] vs. 81% [17/21]). Interestingly, for CE-marked AspLFD, the sensitivity of the test was lower as compared to the prototype. The overall sensitivity of the CE-marked test in BALF samples and serum samples were 64% [98/152] vs. 20% [10/51] [119].

## 5. Parasitic Pneumonia

Due to better hygiene practices and improved socioeconomic conditions, parasitic infestations have declined in the past decade. Nonetheless, the increase in urbanization, the number of immunocompromised patients, and in international travel and global warming has led to a possible rise in the world population that is susceptible to parasitic diseases [134].

*Toxoplasma gondii* and *Strongyloides stercoralis* are the main parasites responsible for pulmonary infections and are associated with high mortality if left untreated in immunocompromised patients [31]. In spite of the high prevalence of parasitic diseases in tropical and developing nations, only a few cases of parasitic pneumonia have been reported in HIV-positive patients [134,135]. In the pre highly active antiviral therapy era, pulmonary toxoplasmosis accounted for only 4% of all pneumonia cases in AIDS patients, while currently it is extremely rare [136]. Also, *Strongyloides stercoralis*, which is common in tropical and subtropical areas, has been reported only occasionally as the cause of pneumonia in AIDS patients [135].

Diagnosing a parasitic infection of the airways can be challenging due to the wide range of clinical presentations, which are frequently nonspecific, and its rarity [134]. Standard microbiological methods, non-invasive antigen testing, and molecular testing can be used.

Disseminated toxoplasmosis has no specific sign or symptoms; fever is frequently present, and various organs, including lungs and central nervous system could be affected. Very few cases of isolated pulmonary involvement mimicking interstitial pneumonia, cytomegalovirus pneumonia, or PJP pneumonia were reported [31]. Diagnosis of toxoplasmosis is mainly based on the detection of protozoan parasites in the body tissue or PCR in blood and/or BALF [137]. Non-invasive methods such as serological testing are unreliable for the diagnosis of toxoplasmosis in immunocompromised transplant patients, as they document only the previous exposure and the risk of reactivation [138].

Currently, cases of *Toxoplasma* spp. pneumonia mainly involved transplant recipients; although, it is very rare. Indeed, a study involving 46 centers from 11 countries in the years 2010–2014 reported 87 toxoplasmosis cases: 58 in allogenic HCT and 29 in SOT recipients, while the average of transplant procedures per country was 1016 for allogenic HCT, 1524 for autologous HCT, and 155 for SOT recipients. Overall, 42 of 87 patients had severe manifestations including pulmonary, cerebral, and disseminated toxoplasmosis; 14 patients had mild manifestations (ocular toxoplasmosis and fever), while 31 had no clinical symptoms. Molecular, serological, culture, and imaging diagnostic method were used, and PCR was the most sensitive (89% [77/89]). In pulmonary toxoplasmosis, PCR was positive in 89% (17/19) of patients, whereas the sensitivity of the serological method (47% [9/19]) and microscopy (32% [6/19]) was lower [139]. Another prospective study included BALF samples from immunocompromised patients over a period of 2 years and compared the diagnostic performance of conventional staining and PCR for pulmonary toxoplasmosis. A total of 336 samples were collected, and two cases of pulmonary toxoplasmosis were diagnosed: one patient had lymphocytic lymphoma, and the other was admitted to the ICU due to IA that developed 7 days after the second liver transplantation [140]. Both cases were positive by PCR and conventional staining, and no other positive results were observed, demonstrating that in the hands of experienced parasitologists, properly performed conventional staining can have similar performance to PCR [140].

There are limited or no data available on the diagnostic performance of the microbiological and parasitological tests for the diagnosis of *Strongyloides stercoralis* pneumonia as the pulmonary infections are uncommon. *Strongyloides stercoralis* is an intestinal parasite, and a definitive diagnosis of pneumonia involves identifying the parasite in respiratory specimens [134,141]. A single-center retrospective study conducted over a period of 10 years (2004–2014) included 16 cases of severe *Strongyloides stercoralis* infection, and 15 of them had pulmonary manifestations. Although no formal assessment of diagnostic methods was performed, all cases were diagnosed by microscopic examinations of larvae through the Agar plate culture method and histopathology [142]. BALF and sputum can be used for the diagnosis of *Strongyloides stercoralis* hyperinfection syndrome when filariform larvae may be found in these fluids [31,143].

## 6. Metagenomics and CRISPR/Cas13a as a Possible Future Approach for the Diagnosis of Severe Pneumonia in Immunocompromised Patients

Metagenomics (mNGS) and metatranscriptomic techniques have emerged as promising diagnostic tools for pneumonia in immunocompromised patients. Nucleic acid (DNA or RNA) from respiratory samples is extracted first and then sequenced with or without PCR amplification, classified, and interpreted. Since mNGS is taxonomically agnostic, no matter what their phylogeny is, pathogens can be identified (bacteria, viral, fungal, and protozoal). A mNGS approach could also help identify resistance genes rapidly [3]. The host–pathogen interaction can also be studied through mNGS, but it may require further research for a correct evaluation and understanding of potential clinical application [144].

A retrospective study assessed the diagnostic performance of BALF mNGS compared with culture-based methods in 69 immunocompromised patients with suspected pneumonia [144]. Overall, mNGS showed higher sensitivity than culture-based methods for all evaluated pathogens (bacterial, viral, and fungal pathogens). Briefly, the sensitivity of mNGS was 93% [95% CI 70–100] for viruses, followed by 91% [95% CI 79–98] for fungi, and 80% (95% CI 63–92) for bacteria. In comparison, the sensitivity of conventional microbiological tests was 53% for viruses, followed by 50% for fungi, and 36% for bacteria, with significantly higher diagnostic accuracy for PJP [145].

A meta-analysis assessed mNGS diagnostic performance in immunocompromised patients with PJP, reporting 96% (95% CI 90–99) and 96% [95% CI 92–98] pooled sensitivity and specificity. Interestingly, the subgroup analysis revealed equally very good performance in BAL and blood samples: sensitivity and specificity in BALF of 94% (95% CI, 78–99) and 96% [95% CI 88–99] and in blood of 93% [95% CI 80–98] and 98% [95% CI 76–100]), respectively [146]. Another meta-analysis reported that the pathogen detection rate was higher for mNGS (80.4% [1233/1532]) than for culture-based methods (45.7% [705/1540]) in patients with severe pneumonia, with a potential favorable effect on mortality rates and length of ICU stay [147].

A turnaround time within 6 h (sample received to pathogens’ identification time) has been reported for the diagnosis of bacterial respiratory tract infections by means of mNGS [148]. A similar turnaround time was also registered for the identification of mycobacterial species, including *Mycobacterium tuberculosis* [149,150]. Furthermore, a retrospective study reported that mNGS was less affected by prior antimicrobial use than the traditional culturing method [151]. Nonetheless, there are some important limitations of mNGS, like clinical enactment, which could be difficult due to of bioinformatics analysis requirements and other issues, such as the difficult differentiation between true infections and colonization, that still deserve further investigation [3,144].

CRISPR/Cas is a self-defense system in prokaryotes, such as bacteria and archaea, and is widely used as a genome editing tool, but more recently, it was applied also as a molecular diagnostic method in different clinical settings [152]. A retrospective study published in 2022 evaluated the diagnostic performance of a CRISPR/Cas13a-based method (RapidCasD) in BALF samples taken from patients suspected of having PJP (19 PJP and 43 non-PJP patients), and 52 patients were immunocompromised. The method achieved 79% [15/19] sensitivity and 98% [42/43] specificity for diagnosing PJP pneumonia, while all the PJP samples were positive by qPCR, with a sensitivity of 100% [19/19] and specificity of 65.1% [28/43]. The RapidCasD methods showed excellent agreement with the clinical diagnosis of PJP as compared to qPCR and suggested the possibility of using this test to differentiate infection from colonization [153]. The CRISPR/Cas13a technique in BALF samples has been also studied for rapid diagnosis of invasive *A. fumigatus* aspergillosis [154]. Finally, another study used a PCR-CRISPR assay to evaluate the performance of CRISPR/Cas13 for the detection of carbapenem-resistant Klebsiella pneumoniae (CRKP). Sixty-one clinical isolates were collected (51 were CRKP and the remaining ten were carbapenem-sensitive, including *P. aeruginosa*, *E. coli*, and *A. baumannii*). The sensitivity and specificity of the PCR-CRISPR Cas13a assay with a fluorescence readout were 92% [47/51] and 100% [0/10] [155].

## 7. Conclusions

In conclusion, microbiological diagnosis of pneumonia in immunocompromised patients remains a challenging and evolving process due to the variety of potential pathogens (Table 1) and the limitations of traditional culture-based diagnostic methods. The combination of molecular methods and antigen testing allows for the early and accurate detection of pneumonia in this vulnerable population. The development of more rapid, sensitive, and cost-effective methods, as well as their validation in a variety of clinical settings, will require further research in this specific population.

## Figures and Tables

**Table 1:** Common etiological agents of pneumonia in immunocompromised patients.

Etiological Agent	Commonly Reported Populations at Risk/Risk Factors	Tests Commonly Used for Diagnosis in Clinical Practice	References
*Legionella*	SOT, HIV, corticosteroid use, HCT	UAT, Culture (BALF, EA, sputum), PCR (BALF, EA, sputum)	[156,157]
*Haemophilus* spp.	Humoral immunosuppression	Culture (BALF, EA, sputum), PCR (BALF, EA, sputum)	[157,158]
*Klebsiella pneumoniae*	All types of immunosuppression	Culture (sputum, blood), PCR (blood, BALF, EA)	[158,159]
*Streptococcus pneumoniae*	Humoral immunosuppression, cancer, SOT, LLC, MM	UAT, culture (BALF, sputum, blood), PCR (sputum, BALF)	[160,161]
*Staphylococcus aureus*	SOT, chemotherapy, chronic lung disease	Culture (blood, sputum, EA, BALF), PCR (BALF, EA, sputum)	[157,159,162]
*Pseudomonas aeruginosa*	COPD, HM, HCT, cancer, cystic fibrosis, chronic kidney disease, HIV, SOT	Culture (sputum, EA, BALF), PCR (BALF, EA, sputum, blood)	[159,163]
*Escherichia coli*	HIV, cancer, SOT, COPD, diabetes mellitus	Culture (BALF, EA, sputum), PCR (BALF, EA, sputum, blood)	[157,164]
*Nocardia* spp.	HIV, SOT, cancer, chronic corticosteroid use, HCT	Culture (sputum, EA, blood, tissue biopsy, BALF), PCR (BALF, sputum)	[157,165,166]
*Mycobacterium tuberculosis*	All types of immunosuppression, particularly anti-TNF-alfa treatment, SOT	Microscopy (sputum smear), culture (BALF, sputum), PCR (sputum, BALF, lungs biopsy)	[167,168]
Cytomegalovirus	HCT, SOT, HIV, alemtuzumab, CLL	PCR (blood, BALF), histopathology	[31,169,170]
Varicella-Zoster virus	HIV, cancer, SOT, chronic corticosteroid use	PCR (BALF, sputum, blood), serology, DFA	[31,171,172]
Herpes simplex virus	SOT, neutropenia, chronic corticosteroid use, ICU patients	PCR (blood, BALF), serology	[31,173]
Influenza, Respiratory syncytial virus (RSV), Coronaviridae (SARS-CoV-2)	HCT, SOT, HM	PCR (BALF, nasopharyngeal, sputum), serology	[31,174,175]
*Aspergillus* spp.	HCT, SOT, neutropenia and corticosteroid therapy	Direct visualization, culture, GM (BALF, serum), PCR (BALF, sputum, blood)	[64,85,176]
*Pneumocystis jirovecii*	SOT, HM, HCT	Direct visualization, BDG (serum), PCR (BALF, sputum, blood)	[97,177,178]
*Cryptococcus* spp.	HCT, SOT	Microscopy, culture (blood, sputum), antigen (serum, BALF, sputum), PCR (BALF, blood)	[31,64,79,179]
*Mucorales* spp.	HM, HCT	Microscopy, culture, histopathology, immunohistochemistry	[31,82,180]
*Toxoplasma* spp.	SOT, HCT, HIV	Microscopy, serological assay, PCR	[137,172,181]
*Strongyloides stercoralis*	SOT, HTLV-1, HIV, HM	Serological methods, and PCR	[182,183,184]

BALF: bronchoalveolar lavage fluid; BDG: 1, 3 beta d glucan; COPD: chronic obstructive pulmonary disease; DFA: direct fluoresce antibody; EA: endotracheal aspirate; HCT: hematopoietic cell transplant; HM: hematological malignancies; HTLV-1: human T-cell lymphotropic virus type 1; HIV: human immunodeficiency virus; LLC, chronic lymphocytic leukemia; MM, multiple myeloma; NAATs: nucleic acid amplification test; PCR: polymerase chain reaction; SOT: solid organ transplant; TA: tracheal aspirate; UAT: urine antigen test.

## Data Availability

No new data were generated.
